# Enhancing Biomedical Text Summarization Using Semantic Relation Extraction

**DOI:** 10.1371/journal.pone.0023862

**Published:** 2011-08-26

**Authors:** Yue Shang, Yanpeng Li, Hongfei Lin, Zhihao Yang

**Affiliations:** School of Computer Science and Technology, Dalian University of Technology, Dalian, Liaoning, China; University of Georgia, United States of America

## Abstract

Automatic text summarization for a biomedical concept can help researchers to get the key points of a certain topic from large amount of biomedical literature efficiently. In this paper, we present a method for generating text summary for a given biomedical concept, e.g., H1N1 disease, from multiple documents based on semantic relation extraction. Our approach includes three stages: 1) We extract semantic relations in each sentence using the semantic knowledge representation tool SemRep. 2) We develop a relation-level retrieval method to select the relations most relevant to each query concept and visualize them in a graphic representation. 3) For relations in the relevant set, we extract informative sentences that can interpret them from the document collection to generate text summary using an information retrieval based method. Our major focus in this work is to investigate the contribution of semantic relation extraction to the task of biomedical text summarization. The experimental results on summarization for a set of diseases show that the introduction of semantic knowledge improves the performance and our results are better than the MEAD system, a well-known tool for text summarization.

## Introduction

The volume of biomedical literature is growing rapidly in recent years. Currently, the number of articles indexed in PubMed is over 19 million. The huge text collection brings a big challenge for human experts to find the information they need. The technique of automatic text summarization can grasp the general information and key points of a certain topic and make the process of knowledge discovery efficient. For example, biologists often need to find the general information about a biological concept, e.g., a gene or a disease, from multiple documents without reading all sentences within the full-text. In this case, an accurate text summarization system can be greatly helpful.

Most existing works on biomedical text summarization focus on using information retrieval (IR) techniques together with domain-specific resources to extract relevant sentences from documents [1]–[8]. In these methods, sentences are ranked and selected based on the similarity measure derived from the overlapping words or concepts between sentences and queries. Luhn *et al.* (1958) [1] develop a text summarization system which selects relevant sentences and generates text abstracts from biomedical literature based on term frequencies. Several methods for sentence ranking consider different weights for texts in different locations of the document, such as sentence position in sections [2], the presence of certain cue phrases [3], and words in title [4]. The MEAD system [5] extracts text summary from multiple documents based on features of position, frequency, and documents cluster centroid. Reeve *et al.* (2007) [6] use the frequency of domain concepts to identify important parts of an article, and then use the resulting concept chains to extract candidate sentences. Ling *et al.* (2006) introduce a gene summary system [7], [8] that extracts information on six aspects of a gene, such as gene products, DNA sequence, etc. In their system, sentences are ranked according to a) the relevance to each aspect of the gene; b) the relevance to the documents where they are from; c) the locations of the sentences within documents. In these approaches, the design of the similarity function for sentence ranking has a big impact on the summarization performance. However, these methods for similarity calculation are only at a word or concept level rather than a semantic-level, since they measure the similarity merely based on the common words or concepts in the query and sentence, which is the major difficulty that limits the performance improvement for text summarization system. For example, in the sentence “The detection of mutation at codon Ser81 of the gyrA gene suggested the potential of developing fluoroquinolone resistance among S. pneumoniae isolates in Malaysia”, the co-occurrence of “codon Ser81 of the gyrA” and “pneumoniae” does not indicate a semantic relationship between them, so the sentence should not be included in the text summary of “pneumoniae”.

Fiszman et al.(2004) [9] apply the technique of information extraction (IE) to extract the entities and relations that are most relevant to a given biological concept from MEDLINE records, and generate a “semantic-level” summary for each concept. Workman *et al.* (2010) [10] apply the method [9] to extract genes relevant to genetic etiology of disease from biomedical literature to support genetic database curation. Workman *et al.* (2011) extend the strategy [9] and develop three statistical methods [11] to automatically identify salient data in bibliographic text and generate summaries for bibliographic based data. Compared to the classical IR-based methods, these methods are able to extract semantic knowledge from biomedical texts and utilize them to generate text summary in a higher-level. However, these applications [9]–[11] differ from the traditional text summarization task[1]–[8], since they cannot generate a reader-friendly summary in plain text, and are not evaluated using the classical evaluation metrics for text summarization such as the method [12]. So it is interesting to see whether the introduction of semantic relation extraction can improve the performance of traditional text summarization system based on classical IR-based methods.

Addressing the problems, in this work we combine the two strategies, i.e. IR and IE based methods, to generate text summaries for biomedical concepts from multiple documents. We aim to examine whether the technique of domain-specific relation extraction can improve the performance of biomedical text summarization. The system consists of three major stages: 1) We extract semantic relations in each sentence using the semantic knowledge representation tool SemRep. 2) We develop a relation-level retrieval method to select the relations most relevant to each query concept and visualize them in a graphic representation. 3) For relations in the relevant set, we extract informative sentences that can interpret them from the document collection to generate text summary using an information retrieval based algorithm. The task in this work is to generate text summaries for a set of diseases from multiple biomedical documents. We evaluate the performance of the system by comparing the automatically generated summary against the textual description of each disease in Wikipedia given by human experts. We examine the contribution of the semantic knowledge and compare our method with some other classical ones for text summarization.

## Methods

**Figure 1:**
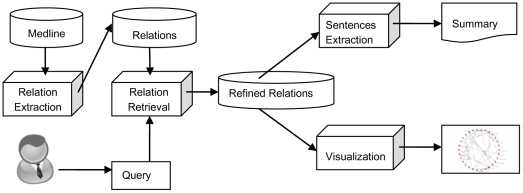
Framework of the biomedical text summarization system.

In this section, we present in detail the three stages i.e., semantic relation extraction, relation retrieval and sentence retrieval, in the text summarization system. In the task of semantic relation extraction, biological concepts and relations between them are extracted from each sentence in the document collection (a subset of MEDLINE abstracts). Relation retrieval aims at selecting the most relevant relations for each query concept from the predictions given by relation extraction. The retrieval algorithm is based on the frequency of relations and semantic types of concepts in the relations. In the final stage, the most relevant sentences that can be used to interpret each relation are extracted from the document collection as the final text summary. The system architecture is illustrated in Figure 1.

### Semantic Relation Extraction

**Figure 2:**
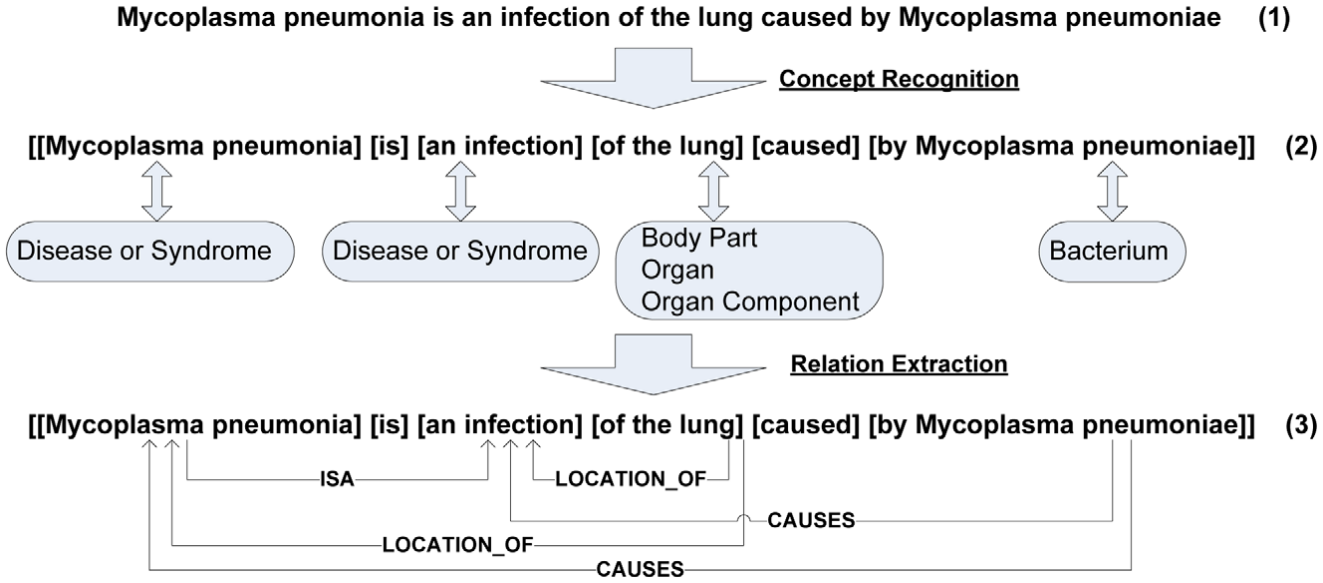
An example of semantic relation extraction.

In this task, we first recognize the biological concepts in each sentence and then extract the relations between them. Figure 2 shows an example of the whole process, where the noun phrases in a sentence are located and mapped into concepts in the UMLS (Unified Medical Language System) Metathesaurus [13], [14], and then the relations between concepts are established using linguistic analysis. Our method is based on the semantic predications provided by SemRep [15], [16], a rule-based symbolic natural language processing system developed by NLM (National Library of Medicine) for biomedical text analysis. The program draws on UMLS information to provide underspecified semantic interpretation in the biomedical domain. The phrase chunking and concept recognition relies on a Xerox part-of-speech (POS) tagger [17], the UMLS Specialist Lexicon [14] and various dictionaries derived from the UMLS Metathesaurus. Simple noun phrases are mapped to concepts in the UMLS Metathesaurus using MetaMap [14]. The relations between the concepts are extracted using syntactic parsing based on dependency analysis and a series of indicator rules which map between syntactic phenomena (such as verbs, nominalizations, and prepositions) and predicates in the UMLS Semantic Network [18].

In a sentence, the relation extracted by SemRep is represented by a triple:

*Rel (Concept1, Predicate, Concept2)*

where *Concept1* and *Concept2* are the two biological concepts that can be found in UMLS Metathesaurus, and *Predicate* is an indicator of the relation type in UMLS Semantic Network. For example, in Figure 2, the triple

*Rel (Mycoplasma pneumonia, CAUSES, infection)*

can be extracted from the context

“…an *infection*… caused by *Mycomplasma pneumonia*.”

In this example, “*infection*” and “*Mycomplasma pneumonia*” are the concepts in Metathesaurus, and “*CAUSES*” is a semantic type in UMLS Semantic Network. In UMLS there is a CUI for each concept in Metathesaurus. For example, “32302” is the CUI of “*Mycoplasma pneumonia*.”

According to the evaluation result reported by NLM [19], for a core set of semantic predicates, such as TREATS, LOCATION_OF, CO-OCCURES_WITH, etc. on a collection of 2000 sentences from MEDLINE, precision and recall on the test collection are 78% and 49% respectively.

### Relation Retrieval

For a query concept there is a large number of sentences with direct relations to the query extracted from the document collection in the first stage. Intuitively, these sentences can be the candidates used to generate text summary due to the semantic relatedness to the query. However, obviously not all these relations can be used to construct the summary, because what users need in practice is usually a short and concise description of the query concept. In addition, some important information tends to be out of the sentences with only direct relations to the query. Addressing these issues, in this stage, we first expand the relations to those that have an indirect relationship with the query, and then select the “most significant” relations from the expanded set based on UMLS Semantic Network and corpus statistics of relations. Note that this search is performed on the annotated texts (the triples predicted by SemRep) in the first stage, which is a higher-level representation than the word-level representation. The advantages are: 1) compared to the classical term-based IR method, the concept-level search can lead to a higher recall without synonym expansion, since the synonyms of noun phrases are recognized and mapped to unique UMLS identifiers during the first stage. 2) The search addresses the semantic relation between the two concepts, thus discarding the sentences where two concepts occur but there is no semantic relationship between them, which can be treated as noise and degrade the retrieval performance. The general steps of algorithm are described as follows:

- Selecting the relations where at least one argument is the query.
- Removing the relations with the frequency under a threshold to generate a core relation set.
- Expanding the set with the new relations that have links to the concepts in the core relation set.
- Removing relations with a “too general” argument.
- Ranking the relations by their frequencies and select top ranked ones as candidates for summary.

In Step a, the relations with direct links to the query are selected, which is the most straightforward way to retrieval concepts and relations relevant to the query. For example, for the query concept “*Angina Pectoris*”, we can get a list of relations such as *Rel (Rose extract, CAUSES, Angina Pectoris)*. More examples in the list are shown in Table 1.

**Table 1:** Direct relations with “Angina Pectoris”.

Angina Pectoris--CO-OCCURS_WITH--Diabetes Mellitus
Rose extract--CAUSES--Angina Pectoris
Blood Platelets--LOCATION_OF--Angina Pectoris
Reduction (chemical)--PROCESS_OF--Angina Pectoris
Angina Pectoris--ISA--Symptoms <2>
Acute hyperglycemia--AFFECTS--Angina Pectoris
Angina Pectoris--CO-OCCURS_WITH--Acute myocardial infarction
Counterpulsation, External--TREATS--Angina Pectoris
Interventions--TREATS--Angina Pectoris
Revascularization - action--TREATS--Angina Pectoris
Exertion--PROCESS_OF--Angina Pectoris
Diabetes Mellitus--CO-OCCURS_WITH--Angina Pectoris
Coronary Artery Bypass--TREATS(INFER)--Angina Pectoris
Angina Pectoris--OCCURS_IN--Male population group
Depressive episode, unspecified--CO-OCCURS_WITH--Angina Pectoris
Angina Pectoris--CO-OCCURS_WITH--Stable angina

**Figure 3:**
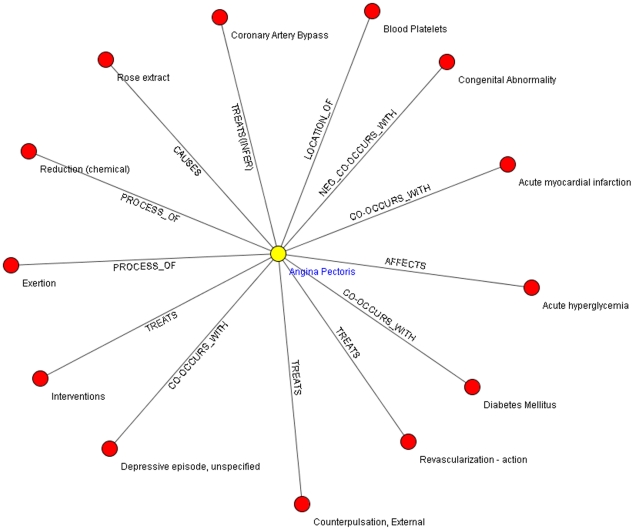
The core relation set for “Angina Pectoris”.

In our experiment, for one disease, at most thousands of relations can be generated in Step a, which makes it difficult for relation expansion and summary generation in the following steps. So in Step b, we select the most important relations for further analysis under the assumption that relations with higher frequency in the document collection tend to be more important. In this step, the frequencies of relations in the text collection are calculated and the ones with the frequency under a threshold are removed. After this filtering, a subset of relations called “core relations” is generated. An example of the semantic relation network after this step is shown in Figure 3.

As is discussed above, sometimes even relations without a direct link to the query can also provide useful information to the users. Step c aims to find the potentially informative relations that can interpret the input concept but do not have direct link to the query concept itself. We apply a simple method that finds all the relations that share an argument with one of the core relations so as to expand the core relation set. For example, the relation “Diabetes Mellitus--CO-OCCURS_WITH--Angina Pectoris” is a core relation for the query “*Angina Pectoris*”. The non-core relation such as “Diabetes Mellitus--CO-OCCURS_WITH--Myocardial Ischemia” is added to the expanded relation set. Relation expansion in this step can improve the recall of the retrieval system greatly, but tends to introduce even more noise than that in Step a. Therefore, the last two steps are designed to remove the noise so as to refine the search result.

Usually, sentences with too general concepts contain little specific information for practical use. For example, the relation “*Pharmaceutical Preparations TREATS Tuberculosis*” cannot give useful suggestions to people who want to know what specific pharmaceutical preparations can treat Tuberculosis disease. In Step d, the argument with a distance less than a threshold from the root concept in MeSH (Medical Subject Headings, see http://www.ncbi.nlm.nih.gov/mesh) is considered as too general to be useful, so the relations containing it are removed from the relation set. MeSH (http://www.ncbi.nlm.nih.gov/mesh) is one of the most widely used Ontology resources in biological domain constructed by human experts. It has a tree-style concept hierarchy, where the concepts close to the root of tree tend to be general concepts. Here the distance refers to the number of edges to the root node. For example, the distance for the root node itself is 0, and for nodes in the second layer is 1. In the experiment section, the impact of different distances (from 0 to 17) from the root will be investigated. The distance used in the final system is 3.

**Figure 4:**
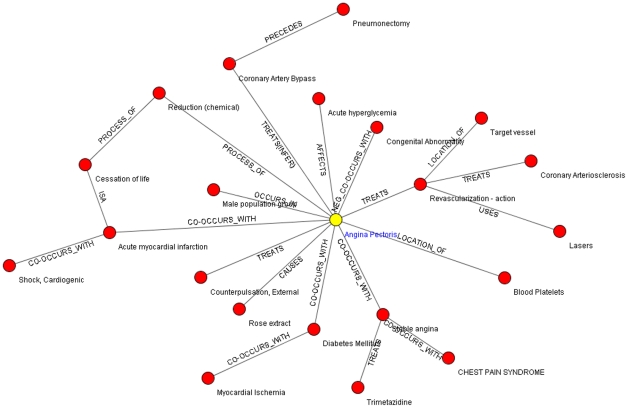
Semantic relation network for “Angina Pectoris” after relation retrieval.

Similar to Step b, Step e also calculates the frequency of relations and selects the ones occurring more frequently than an empirically determined threshold value. The final network of the refined relation set is shown in Figure 4.

After the whole process of the relation retrieval, a relation-level summary together with a graph representation is generated for a given query concept. The semantic relation graph can help users to get the general knowledge of the query concept, but it does not provide specific description in natural language, so we need to extract sentences to support each relevant relation to generate summary in plain text in the following stage.

### Sentence Retrieval

In this stage, for each query, we rank and select the most relevant sentences for each relation obtained in the relation retrieval to generate the final text summary. Sentence retrieval based on information retrieval (IR) techniques is the classical method for text summarization in various domains [3], [20], [21].Our method differs from other IR based approaches for text summarization in the following aspects: 1) the selection of sentences is based on the output of relation retrieval, which means that the method combines the relation retrieval and sentence retrieval to get a better performance on text summarization. The goal of this research is to investigate whether the use of relation extraction can improve the performance of classical IR based method for text summarization. 2) Considering the different importance of sentences in different regions in the document e.g., Title, Abstracts, and Background, our method assigns different weights to them.

In addition, diversity is an important measure to evaluate IR systems[22]–[24]. Usually high diversity in the retrieved result is preferred, which encourages the search results describe the query in different aspects. Since the sentences are extracted from different relations, intuitively the two-stage retrieval method can obtain a higher semantic diversity for the sentences in the final summary than word-based search. For example, word-based search may lead to the result where many sentences (possibly all) in the summary are derived from the same relation, but relation-based search can avoid the case. However, in the final summary there may be redundant sentences retrieved within each relation or across the relations with high semantic similarity. So we design a post-processing method to remove the redundant sentences in the candidate set for text summarization. The method for sentence retrieval can be divided into two steps: sentence ranking and redundant sentence removal.

### Sentence Ranking

**Figure 5:**
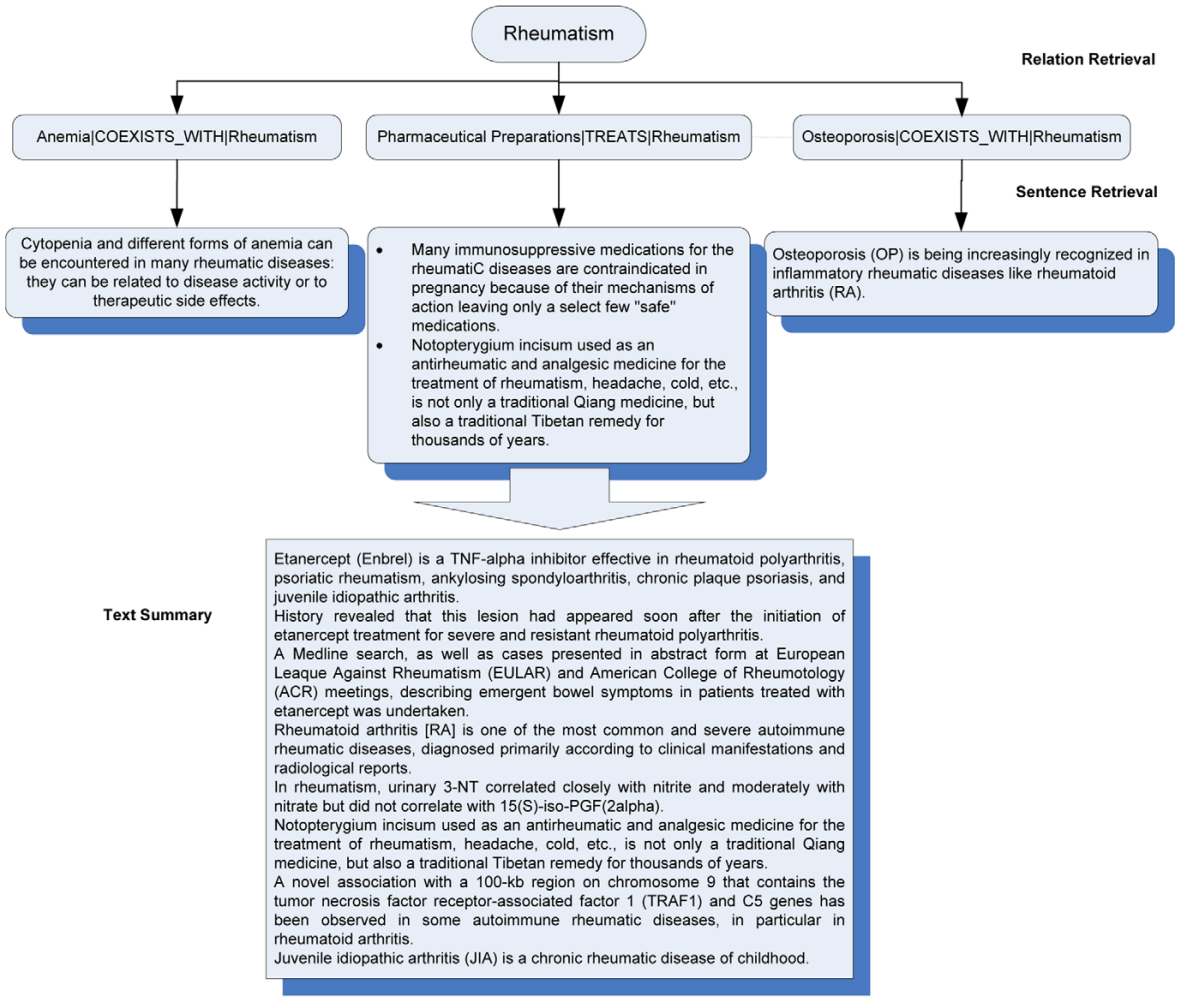
An example of relation and sentence retrieval.

We use Okapi BM25 [25], one of the most prevailing IR techniques, to rank sentences that contain the relations extracted in the relation retrieval. An example of both the relation and sentence level retrieval is shown in Figure 5.

Given a relation *R*, containing key words *r_1_*, *r_2_*,……, *r_n_*, the BM25 score of a sentence *S* is computed as:
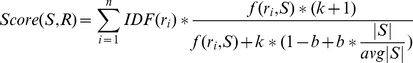
(1)
*f(r_i_,S)* is the frequency of *r_i_* in sentence *S*, *|S|* is the length of sentence *S* in words, and *avg|S|* is the average sentence length in the sentence collection. *k* and *b* are free parameters, usually chosen as *k* = 2.0 and *b* = 0.75.

*IDF(r_i_)* is computed as formula(2):
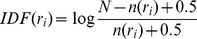
(2)
*N* is the total number of sentence in the collection, and *n(r_i_)* is the number of documents containing *r_i_*.

In biomedical literature, sentences in some sections are more suitable for text summary than other sections. For example, texts in TITLE, BACKGROUND, or CONCLUSION usually contain more general descriptions of certain topics than the EXPERIMENT section, so the location of the sentence can be used as the cue to rank sentences if the BM25 score cannot work well. Let *Score_Loc_* be the location score. For a relation *R* and a sentence *S*, we combine the BM25 score and location score via the following formula to give the final score of the sentence:
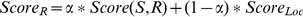
(3)where 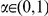 is the trade-off parameter that is used to balance the impact of BM25 score and location score, which is obtained by 5-fold cross validation on the dataset and the impact of parameter selection will be discussed in the “Results and Discussions” section. The section and location scores used in our experiment are listed in Table 2. For each relation, we rank the sentences by the final score and select the top ranked 5 sentences as the candidate set for text summaries.

**Table 2:** Location scores used in our experiment.

Location Tag	Location Score
BACKGROUND	1
CONCLUSIONS	
TITLE	
OBJECTIVES	
MATERIAL AND METHODS	0.5
RESULTS	

### Redundant Sentence Removal

We use the method [26] to remove redundant sentences in the retrieved results of sentences ranking. The general idea is to penalize the candidate sentences that have high similarity with the ones already in the summary. Assume that *S* is the set of sentences in the final summary and *C* is the set of candidate sentences. The algorithm process is as follows:

(1) Initialize the two sets *S* = ∅ and 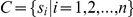 containing all the extracted sentences. Initialize the score of each sentence in *C* using Formula (3).

(2) Sort the sentences in *C* based on the scores of sentences.

(3) Select the top ranked sentence 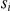 in *C*. Move 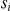 from *C* to *S* and update the score of each remaining sentence *j* in *C* as follow:
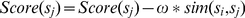
(4)where 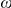
>0 is the penalty factor for diversity, and 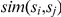
is the cosine similarity between 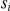
and 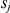.

(4) Repeat step (2) and step (3) until the number of selected sentences reach the summary length.

In our experiment, the parameter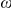 is assigned at 3, which is also obtained by cross-validation and relevant experimental results will be shown in “Results and Discussions” section. The length of final summary is fixed at 8 sentences.

## Results and Discussions

### Experimental Design

It is well known that the evaluation of text summarization is extremely difficult in IR domain even for human being, since different users may be interested in different aspects of the query in different applications, thus leaving much flexibility to determine the accuracy of a text summary. Existing evaluation approaches for text summarization relies on comparing the text summary generated by computer with a “gold standard” (reference summary) given by human experts [12]. The task for examining our approaches is to generate text summaries for a set of diseases (Table 3).

**Table 3:** Diseases use in our experiment.

Alzheimer's Disease	Cerebrovascular accident	Epilepsy	Myocarditis
Asthma	Colon Carcinoma	HIV Infections	Myotonic Dystrophy
Atherosclerosis	Crohn's disease	Huntington Disease	Obesity
Breast Carcinoma	Cystic Fibrosis	Hypertensive disease	Schizophrenia
Carcinoma of lung	Depressive disorder	Malaria	Parkinson Disease
Cerebral Amyloid Angiopathy	Mad Cow Disease	Metabolic syndrome	Prostate carcinoma

We make use of the definition in Wikipedia for each disease as the reference summary and evaluate the performance based on the overlap of the summary generated by our system and the reference summary in Wikipedia. Our document collection is a subset of the MEDLINE abstracts of the year 2009, which covers 500,493 biomedical research papers with 1.7 million sentences.

We use the ROUGE evaluation package as evaluation metric [12]. ROUGE is a recall-based metric for fixed-length summaries which is based on n-gram overlap. Among ROUGE metrics, ROUGE-N (models n-gram co-occurrence, N = 1, 2) and ROUGE-L (models longest common sequence) generally perform well in evaluating both single-document summarization and multi-document summarization [27]. Thus although we evaluated our methods with all the metrics provided by ROUGE, we only report ROUGE-1, ROUGE-2 and ROUGE-L in this paper (other metrics give very similar results). In order to truncate summaries longer than length limit, we used the “-l” option in ROUGE toolkit. We also used the “-m” option for word stemming. We take ROUGE-1 for example to illustrate how ROUGE package works.
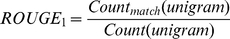
(5)
*Count_match_(unigram)* is the maximum number of n-grams co-occurring in a reference summary and a model unit. *Count (n-gram)* is the number of n-grams in the model unit.

In the experiment, we examine the performance of our summarization system in the following aspects:

1. We compare the performance of our system with two baselines: the method without using relation retrieval and the MEAD system [5], a well-known system for text summarization for general domain.
2. We examine the contribution of relation expansion (Step c) and noise filtering (Step d) in relation retrieval, as well as the method for redundant sentence removal and the impact of parameter selection in several components of the system.

### Comparison of Summarization Approaches

To our best knowledge, currently there is no system publicly available for biomedical text summarization, so we compare our system with two classical text summarization methods. To examine the impact of semantic information, we design a baseline (named N_SR) which ranks and selects sentences only based on the combination of BM25 score and location score (in the “Sentence Ranking” section) and removes the redundant sentences (in the “Redundant Sentence Removal” section) without taking semantic relations into account. The second one is a well known publicly available summarizer – MEAD [5]. We use the latest version of MEAD 3.11 with default setting. We test our text-based summary by ROUGE metric.

**Figure 6:**
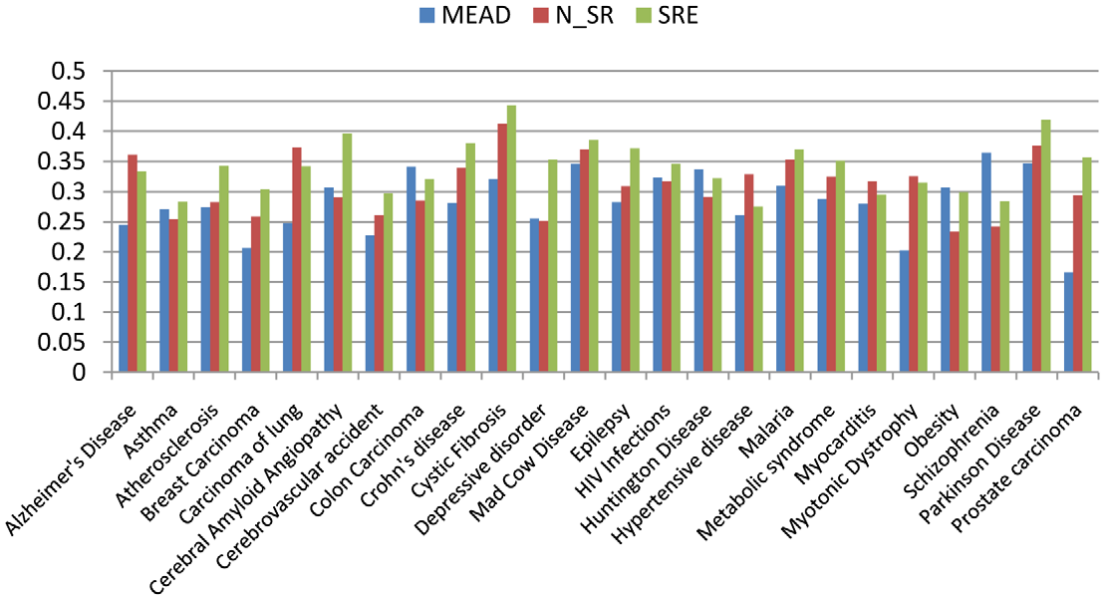
Comparison of summarization performance on ROUGE-1.

Table 4 and Figure 6 show the comparison of these methods on ROUGE-1, ROUGE-2 and ROUGE-L for each disease as well as the average performance. We can see that the method based on semantic relation extraction, short for SRE, outperforms the baselines in most cases. To examine the statistical significance of the improvement, we perform t-test on the result set “N_SR vs. SRE” and “MEAD vs. SRE”. The p-values are 0.00 and 0.001 respectively for the two pairs. Usually a p-value less than 0.05 is considered as statistical significance. When analyzing the results, we find that the reference summaries from Wikipedia usually contain biomedical semantic information such as “cause”, “treatment” and “pathogenesis”, which is well addressed in our method for semantic extraction and retrieval but not in the classical text summarization methods (N_SR and MEAD) which extract sentences only based on keyword matching without considering whether there is a semantic relation between the co-occurrence concepts. Therefore, these results show that the introduction of semantic knowledge has contribution to improve the performance of biomedical text summarization. The MEAD system uses a set of heuristics based on keyword matching and Ontology mapping without considering semantic relation either. The results in Table 4 show that the method N_SR outperforms the MEAD system on average. The difference in performance reflects the comparison of the heuristics used in MEAD system and the BM25 algorithm plus location score and the redundancy removal used in our experiment. The results indicate that classical IR-based method (N_SR) considering location information and redundant removal is more effective than (at least as well as) the heuristic-based method (MEAD) in this task.

**Table 4:** Performance of summarization for 24 diseases.

Disease	ROUGE-1			ROUGE-2			ROUGE-L		
	MEAD	N_SR	SRE	MEAD	N_SR	SRE	MEAD	N_SR	SRE
Alzheimer's Disease	0.2446	0.3613	0.3333	0.0431	0.0509	0.0518	0.1785	0.2968	0.2910
Asthma	0.2708	0.2542	0.2828	0.0310	0.0389	0.0684	0.2154	0.2542	0.2239
Atherosclerosis	0.2738	0.2825	0.3424	0.0147	0.0426	0.0511	0.2103	0.2429	0.2420
Breast Carcinoma	0.2062	0.2584	0.3040	0.0195	0.0259	0.0260	0.1479	0.1912	0.1888
Carcinoma of lung	0.2475	0.3731	0.3418	0.0441	0.0570	0.0828	0.1916	0.2994	0.2799
Cerebral Amyloid Angiopathy	0.3067	0.2905	0.3963	0.0604	0.0966	0.1067	0.2733	0.2849	0.2945
Cerebrovascular accident	0.2269	0.2606	0.2974	0.0539	0.0375	0.0601	0.2000	0.2359	0.2279
Colon Carcinoma	0.3414	0.2852	0.3208	0.0264	0.0300	0.0318	0.2845	0.2257	0.2692
Crohn's disease	0.2810	0.3393	0.3806	0.0282	0.0526	0.0620	0.2576	0.2828	0.3188
Cystic Fibrosis	0.3206	0.4122	0.4432	0.0769	0.1176	0.1176	0.2632	0.3461	0.3562
Depressive disorder	0.2554	0.2514	0.3527	0.0399	0.0501	0.0445	0.2289	0.2329	0.3328
Mad Cow Disease	0.3455	0.3698	0.3852	0.0857	0.1042	0.0838	0.3090	0.3340	0.3737
Epilepsy	0.2820	0.3086	0.3718	0.0158	0.0544	0.0517	0.2350	0.2914	0.3243
HIV Infections	0.3232	0.3172	0.3458	0.0753	0.0617	0.0462	0.2764	0.2943	0.3223
Huntington Disease	0.3366	0.2910	0.3218	0.0547	0.0390	0.0436	0.3168	0.2601	0.2913
Hypertensive disease	0.2609	0.3284	0.2751	0.0328	0.0388	0.0677	0.2283	0.2687	0.2424
Malaria	0.3093	0.3529	0.3699	0.0259	0.0836	0.0807	0.2680	0.3476	0.3429
Metabolic syndrome	0.2878	0.3249	0.3509	0.0580	0.0811	0.0727	0.2086	0.2888	0.2483
Myocarditis	0.2795	0.3171	0.2952	0.0570	0.0590	0.0735	0.2445	0.3024	0.2889
Myotonic Dystrophy	0.2023	0.3253	0.3146	0.0528	0.0714	0.0606	0.1873	0.2831	0.3121
Obesity	0.3067	0.2333	0.2990	0.0253	0.0383	0.0200	0.2647	0.2283	0.2924
Schizophrenia	0.3644	0.2419	0.2841	0.0436	0.0338	0.0324	0.2473	0.2043	0.2269
Parkinson Disease	0.3469	0.3757	0.4191	0.0498	0.0703	0.0638	0.3288	0.3333	0.3170
Prostate carcinoma	0.1662	0.2938	0.3567	0.0051	0.0396	0.0503	0.1662	0.2625	0.2670
Average	0.2828	0.3104	0.3410	0.0425	0.0573	0.0604	0.2388	0.2746	0.2864

### Impact of Components and Parameter Selection

Table 5 shows the impact of relation expansion (Step c in the “Relation Retrieval” section), noisy filtering (Step d in “Relation Retrieval”) in relation retrieval and redundant sentence removal. It can be seen that introduction of these steps improves the overall performance of text summarization. The combination of these methods lead to around 0.02–0.03 absolute improvement and 6%–60% relative improvement on the three evaluation metrics for text summarization. These results justify our analysis in previous sections. Note that for simplicity we only observe the performance on ROUGE-1, the most widely used evaluation metric, for the experiments in this section.

**Table 5:** The impact of relation expansion, noise filtering and redundant removal.

Method	ROUGE-1	ROUGE-2	ROUGE-L
Baseline	0.3196	0.0373	0.2693
Expansion	0.3263(+2.0%)	0.0408(+9.4%)	0.2723 (+1.1%)
Filtering	0.3208(+0.4%)	0.0397(+6.4%)	0.2655 (-1.4%)
Expansion + Filtering	0.3303(+3.3%)	0.0436(+16.9%)	0.2801 (+4.0%)
Expansion + Filtering +Redundant Removal	0.3410(+6.7%)	0.0604(+61.9%)	0.2864 (+6.3%)

**Figure 7:**
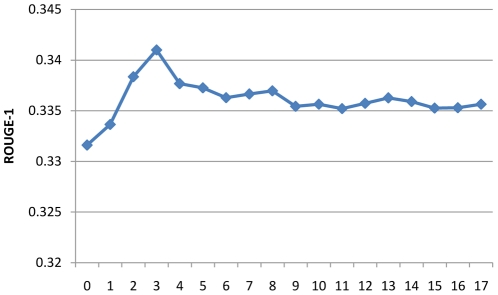
Relationship between ROUGE-1 and concept depth in MeSH based filtering.

**Figure 8:**
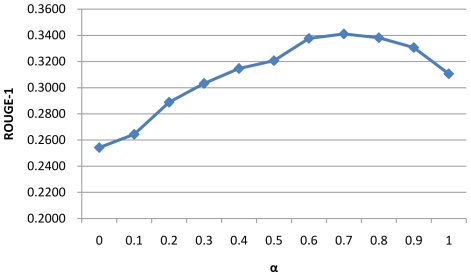
Relationship between ROUGE-1 and the trade-off parameter 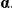

**Figure 9:**
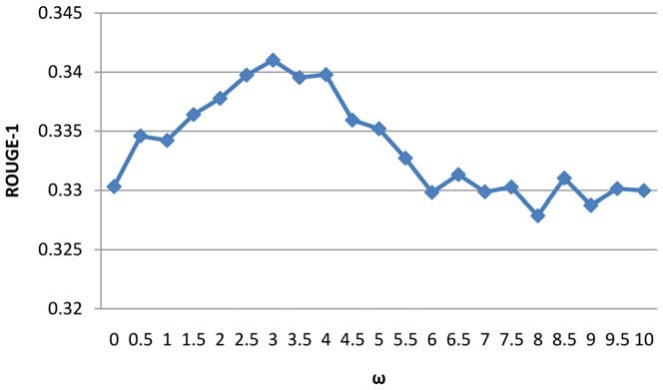
Relationship between ROUGE-1 and the trade-off parameter ω.

Since several parameters are used in the different step process, we design experiments to examine the impact of parameter selection to the summarization performance. We investigate the impact of three parameters: 1) the depth of concept in MeSH used in the noise filtering step (step d in “Relation Retrieval”) in relation retrieval (Figure 7); 2) the trade-off parameter α between BM25 score and location score in Formula 3(Figure 8); 3) the trade-off parameter ω between relevance and diversity in Formula 4 (Figure 9).

From Figure 7 we can see that the optimal concept depth for noise filtering is 3. The performance firstly improves with depth of concept increasing and then decreases, and finally seems to converge to a specific value around 0.335. As discussed in the section “Relation Retrieval”, in the hierarchy structure of MeSH, general concepts in small depth tend to be noises in concept expansion. The lowest performance occurs at the depth of 0, where no concepts are filtered. This run is equal to relation expansion without filtering and its inferior performance reflects the impact of noise in relation expansion. In contrast, when a lot of specific concepts (such as the depth more than 10) are removed from the expansion set, the run is almost equal to the method without concept expansion, which achieves a performance between the optimal method and the unfiltered method.

Figure 8 and Figure 9 show the impact of parameter α and ω in the sentence retrieval stage. It can be seen that the optimal values of α and ω lie in the interval (0.6, 0.9) and (2.5, 4.5) respectively, and the performance is not very sensitive to these parameters in a certain interval.

### Conclusion

In this paper, we present a system for biomedical text summarization based on the techniques of semantic relation extraction and information retrieval. The experimental results demonstrate that the incorporation of semantic knowledge can enhance the performance of text summarization in biomedical domain. Moreover, the semantic relation network generated in our approach is able to help the user for a quick understanding of the query concept.

A text summarization system should be the integration of several key components e.g., shallow parsing, information extraction, information retrieval or semantic similarity design, and its overall performance relies heavily on the individual performance of its components. In the future, we will focus our research on improving the performance of these components. For example, we will develop more accurate algorithm for semantic relation extraction and retrieval, and design semantic similarity measure by integrating information from unlabeled data e.g., the MEDLINE corpus and various semantic recourses e.g., Wordnet or MeSH. In addition, we will extend our method to extract summaries for other biological concepts e.g., genes, proteins or drugs.
